# Opposing patterns in self-reported and measured physical activity levels in middle-aged adults

**DOI:** 10.1007/s10433-021-00657-z

**Published:** 2021-10-29

**Authors:** Jin Luo, Raymond Y. W. Lee

**Affiliations:** 1 School of Biomedical Sciences, University of West London, St Mary′s Rd, London, W5 5RF UK; 2 University of Portsmouth, Portland Building, Portland Street, Portsmouth, PO1 3AH UK

**Keywords:** Ageing, Middle adulthood, Self-reported physical activity, Accelerometry, Bone, Muscle

## Abstract

Physical activity brings significant health benefits to middle-aged adults, although the research to date has been focused on late adulthood. This study aims to examine how ageing affects the self-reported and accelerometer-derived measures of physical activity levels in middle-aged adults. We employed the data recorded in the UK Biobank and analysed the physical activity levels of 2,998 participants (1381 men and 1617 women), based on self-completion questionnaire and accelerometry measurement of physical activity. We also assessed the musculoskeletal health of the participants using the dual-energy X-ray absorptiometry (DXA) measurements provided by the UK Biobank. Participants were categorised into three groups according to their age: group I younger middle-aged (40 to 49 years), group II older middle-aged (50 to 59 years), and group III oldest middle-aged (60 to 69 years). Self-reported physical activity level increased with age and was the highest in group III, followed by group II and I (P < 0.05). On the contrary, physical activity measured by accelerometry decreased significantly with age from group I to III (P < 0.05), and the same pertained to the measurements of musculoskeletal health (P < 0.05). It was also shown that middle-aged adults mostly engaged in low and moderate intensity activities. The opposing trends of the self-reported and measured physical activity levels may suggest that middle-aged adults over-report their activity level as they age. They should be aware of the difference between their perceived and actual physical activity levels, and objective measures would be useful to prevent the decline in musculoskeletal health.

## Introduction

Musculoskeletal health is important for mobility and physical functions of *the human body*. The decline in muscle and bone mass associated with ageing leads to disability, frailty, and reduced quality of life in the elderly (Greco et al. 2019; Kirk et al. 2020). Although osteoporosis and sarcopenia often manifest themselves in later life, the processes leading to them have been shown to start in middle adulthood. The decline in muscle mass starts from around the age of 30 years, but the rate of decline becomes noticeable after 45 years of age (Janssen et al. 2000). Similarly, bone mass peaks at the third decade of life and begins to decrease from 50 years of age. The decrease in bone mass is accelerated for women at the time of menopause (Compston 2001). However, there is evidence that physical activity in middle-aged adults is associated with stronger bones and muscles (Chahal et al. 2014).

Earlier studies show that physical activity in middle-aged adults may reduce the risks of illness and promote independent living in later life (Kelly et al. 2016). However, if people perceive their physical activity level as sufficient during the ageing process, they will be less likely to motivate themselves to engage in more physical activities (Visser et al. 2014). Perception of physical activity level can generally be measured by various types of questionnaires in which subjects report the duration and frequencies of their physical activity. This method has been used by many previous studies to assess physical activities in the general population (Shephard 2003). On the other hand, physical activity can also be measured objectively using motion sensors (e.g. accelerometers) which are attached to the human body. These two methods of measurement often provide different findings about the physical activity level of the subjects. It has been shown that self-reported physical activity tends to overestimate the level of physical activity compared to the objective method (Colley et al. 2018; Dyrstad et al. 2014; Hagstromer et al. 2010). Although the overestimation happens in adults of all age groups, people are more likely do so in late adulthood (Dyrstad et al. 2014), suggesting that ageing may play a significant role in the change of perception of physical activity level. However, there has been little research on how ageing processes change the perception of physical activity in middle adulthood, and how such perception may be different to actual physical activity level which may affect overall life expectancy and the likelihood of chronic diseases during late adulthood (Li et al. 2020).

The aim of the current study was to study the self-reported (perceived) and accelerometer-derived (objective) measures of physical activity level in different age cohorts among middle-aged adults and the associated changes in musculoskeletal health.

## Methods

### Study design and population

The current study is a cross-sectional study based on UK Biobank data. UK biobank is a large long-term study in the United Kingdom on a cohort consisting of around 500,000 UK men and women who were recruited during the period 2006–2010. The current study is based on a sample of 2,998 participants (1381 men and 1617 women) which was drawn from the UK Biobank database. Participants were included if they had participated in all of the following three assessments:

- Physical activity level data collected using self-completed questionnaire,
- Seven-day physical activity data of participants which were collected using a triaxial accelerometer, and
- Musculoskeletal health data were collected from participants using dual-energy X-ray absorptiometry (DXA) scans.

Participants were categorised into three age groups according to their age at the time of recruitment: group I younger middle-aged (40 to 49 years old), group II older middle-aged (50 to 59 years old), and group III oldest middle-aged (60 to 69 years old).

### Information from UK Biobank datasets

#### Participants

Participants’ age, gender, height (standing), weight, and body mass index (BMI) were recorded at initial assessment.

#### Self-reported physical activity level

Self-reported physical activity of the participants was obtained at the time of recruitment from their self-completion questionnaire, in which they were asked to report frequency and duration of physical activities during the last 4 weeks prior to the day of reporting. The questionnaire was adapted from the International Physical Activity Questionnaires (IPAQ) and the Recent Physical Activity Questionnaire (RPAQ), both of which are validated survey instruments (Besson et al. 2010; Craig et al. 2003). The type of physical activities in the questionnaire included walking, walking for pleasure, moderate physical activity, vigorous physical activity, strenuous sports, light do-it-yourself (DIY), heavy DIY, and other exercises. Definitions and/or examples of physical activities were provided to participants to help them categorise their activities into the different types. Participants reported the frequency of a physical activity as number of times per week that they performed the activity. There are two different ways for participants to report the duration, depending on the type of activities. For walking, moderate physical activity, and vigorous physical activity, participants reported the number of minutes spent on each type of these activities per day. For other types of activities, participants chose one from seven categories that was appropriate for them (e.g. 1 = less than 15 min, 2 = between 15 and 30 min, 3 = 30 min and 1 h, 4 = Between 1 and 1.5 h, 5 = Between 1.5 and 2 h, 6 = Between 2 and 3 h, 7 = Over 3 h). The self-reported frequency and duration of physical activities were used for analysis.

#### Measured physical activity level

Physical activity during a 7-day period was measured by a wrist-worn triaxial accelerometer (AX3, Axivity Ltd, UK). The accelerometer was set to sample acceleration data at 100 Hz with a dynamic range of ± 8 g. The quality of the raw acceleration data from each participant had been checked by UK Biobank against two criteria (Doherty et al. 2017). The first criterion was that the participant had at least three days (72 h) of wear data and also had wear data in each 1-h period of the 24-h cycle. Secondly, the raw accelerometer data had to be shown to be well calibrated.

Raw acceleration data from each participant were first uploaded into Open Movement AX3 OMGUI (version 1.0.0.30, Newcastle University, UK) to extract 12-h (8 a.m. to 8 p.m.) long acceleration data from each day. Loading dose of physical activity during the 12-h period was then calculated at four loading intensity (LI) categories using a customised MATLAB program (Mathworks Inc, Natick, MA, USA)—very light (LI < 5 BW/s), light (5 BW/s < LI < 10 BW/s), moderate (10 BW/s < LI < 15 BW/s), vigorous (LI > 15 BW/s). The computational algorithm takes into account of body weight, the magnitude of acceleration signals and the duration of physical activity in deriving the loading dose and has been fully described in our previous publications (Chahal et al. 2014; Kelley et al. 2014). Duration of physical activity spent in a specific intensity category was also calculated for each day. The average value of loading dose and duration of physical activity across the seven days was used for analysis.

#### Musculoskeletal health

DXA measurements (GE-Lunar iDXA, Madison, WI, USA) were conducted on participants to obtain numerical measures of leg leam mass (left side), trunk lean mass, total lean mass, and bone mineral density (BMD) at whole spine (C4 to L4), lumbar spine (L1 to L4), and hip.

### Statistical analysis

Multivariate analysis of variance (MANOVA) was used to examine the differences among the three age groups in self-reported frequency and duration of physical activity, the calculated loading dose and duration of measured physical activity, and musculoskeletal health. Weight, height, BMI, and gender were included in the analysis as covariates.

Correlation analysis was conducted to examine the relationship between self-reported and measured levels of physical activity for each age group and at different intensity level.

SPSS 22.0 (IBM, Armonk, NY, USA) was used to carry out all the analysis. A P value of less than 0.05 is considered to be statistically significant.

## Results

Participants’ weight, height, and BMI were not significantly different among the three groups at the baseline (P > 0.05) (Table 1).

**Table 1:** Participant characteristics at initial assessment

Group	Group		
	I	II	III
Sample size (M/F)	718 (278/440)	1158 (514/644)	1122 (589/533)
Age (yrs)	45.1 (2.8)	54.9 (2.8)	63.5 (2.8)
Weight (kg)	77.1 (16.4)	77.4 (15.7)	76.3 (13.9)
Height (cm)	169.9 (8.7)	169.2 (9.7)	168.9 (8.8)
BMI (kg/m^2^)	26.6 (4.9)	26.9 (4.5)	26.7 (4.0)

Data are presented as mean (S.D.); Group I: 40–49 yrs; Group II: 50–59 yrs; Group III: 60–69 yrs

### Self-reported physical activity level

MANOVA showed that the differences in the frequency [F(16, 5518) = 9.78, P < 0.001] and duration [F(16, 5024) = 9.10, P < 0.001] of self-reported physical activity were statistically significant among the three age groups. Self-reported frequency and duration of physical activity were the highest in group III, followed by group II and I (Tables 2 and 3). Such increases among the age groups were clearly shown in physical activities with low and moderate intensities such as walking, walking for pleasure, moderate activity, and light DIY. Regarding physical activities with high intensities (e.g. vigorous activity, strenuous sports, and heavy DIY), the self-reported physical activity level either increased slightly or decreased with age.

**Table 2:** Self-reported frequency of physical activity (times/week)

Group	Group			df	F	P values
	I (n = 718)	II (n = 1158)	III (n = 1122)			
Walking	5.2 (1.9)	5.3(1.9)	5.5 (1.8)	2, 2764	10.62	< 0.001
Moderate activity	3.3 (2.3)	3.4(2.3)	3.8(2.2)	2, 2764	12.99	< 0.001
Vigorous activity	2.0(1.8)	1.8 (1.8)	1.8 (1.9)	2, 2764	3.21	0.041
Walking for pleasure	2.9 (1.5)	3.1 (1.5)	3.4 (1.4)	2, 2764	19.76	< 0.001
Strenuous sports	3.4 (1.2)	3.5 (1.1)	3.5 (1.1)	2, 2764	30.82	< 0.001
Light DIY	2.8 (1.2)	3.0 (1.3)	3.3 (1.3)	2, 2764	11.22	< 0.001
Heavy DIY	2.3 (1.1)	2.4 (1.1)	2.6 (1.2)	2, 2764	12.82	< 0.001
Other exercise	3.5(1.2)	3.4(1.2)	3.5(1.3)	2, 2764	4.58	0.01

Data are presented as mean (S.D.); Group I: 40–49 yrs; Group II: 50–59 yrs; Group III: 60–69 yrs

**Table 3:** Self-reported duration of physical activity

Group	Group			df	F	P values
	I (n = 718)	II (n = 1158)	III (n = 1122)			
Walking (min/day)	52.4 (70.1)	57.3 (75.3)	53.0 (56.7)	2, 2517	0.986	0.373
Moderate activity (min/day)	45.5(64.6)	47.5(68.1)	59.2(70.4)	2, 2517	8.34	< 0.001
Vigorous activity (min/day)	31.2(44.1)	26.7(36.9)	26.9(40.2)	2, 2517	4.21	0.015
Walking for pleasure	3.6(1.5)	3.8(1.6)	4.1(1.7)	2, 2517	17.61	< 0.001
Strenuous sports	3.6(1.1)	3.7(1.2)	4.0(1.3)	2, 2517	25.99	< 0.001
Light DIY	3.2(1.5)	3.3(1.3)	3.5(1.5)	2, 2517	4.21	0.015
Heavy DIY	3.9(1.6)	3.7(1.6)	3.9(1.7)	2, 2517	7.59	0.001
Other exercise	3.2(1.1)	3.3(1.2)	3.6(1.5)	2, 2517	1.29	0.274

Data are presented as mean (S.D.); Group I: 40–49 yrs; Group II: 50–59 yrs; Group III: 60–69 yrs

### Measured physical activity level

MANOVA demonstrated that the differences in the duration [F(8, 5970) = 9.44, P < 0.001] and loading dose [F(8, 5968) = 8.10, P < 0.001] of measured physical activity were statistically different among the three age groups. However, the duration and loading dose of physical activity level as measured by accelerometry showed the opposite patterns compared with self-reported physical activity. The duration and loading dose of activities with moderate intensity decreased significantly by 22 and 21% from group I to group II (P < 0.05) and decreased further by 28% and 29% from group II to III (P < 0.05) (Table 4). Similarly, the duration and dose of vigorous intensity physical activities decreased significantly by 9% and 12% from group I to II (P < 0.05) and by 43% and 42% from group II to III (P < 0.05). The doses of very light and light intensity physical activity also decreased significantly from group I to III, while the duration of very light intensity physical activity increased (P < 0.05).

**Table 4:** Physical activity measured by accelerometry

Group	Group			df	F	P values
	I (n = 718)	II (n = 1158)	III (n = 1122)			
Duration of VLPA (s)	42,480 (554)	42,555 (525)	42,647 (404)	2, 2985	11.27	< 0.001
Duration of LPA (s)	541 (418)	489 (392)	428 (322)	2, 2985	28.12	< 0.001
Duration of MPA (s)	95 (188)	74 (167)	53 (105)	2, 2985	23.52	< 0.001
Duration of VPA (s)	23 (67)	21 (96)	12 (37)	2, 2985	11.22	< 0.001
Dose of VLPA (BW)	39,083(8917)	38,959(8770)	37,491(7989)	2, 2985	37.49	< 0.001
Dose of LPA (BW)	3519(2825)	3159(2614)	2761(2149)	2, 2985	28.47	< 0.001
Dose of MPA (BW)	1141(2299)	897(2081)	636(1289)	2, 2985	23.94	< 0.001
Dose of VPA (BW)	415(1180)	367(1665)	213(649)	2, 2985	11.07	< 0.001

Data are presented as mean (S.D.); Group I: 40–49 yrs; Group II: 50–59 yrs; Group III: 60–69 yrs; VLPA = very light intensity physical activity (LI < 5 BW/s); LPA = light intensity physical activity (5 BW/s < LI < 10 BW/s); MPA = moderate intensity physical activity (10 BW/s < LI < 15 BW/s); VPA = vigorous intensity physical activity (LI > 15 BW/s); LI = loading intensity; BW = body weight

The accelerometer data also showed that the participants spent most of their time on physical activities at very light, light and moderate levels.

### Musculoskeletal Health

MANOVA showed that the differences in musculoskeletal health [F(18, 4980) = 26.66, P < 0.001] were statistically different among the three age groups. The lean mass and bone mineral density (BMD) of the participants decreased significantly from group I to III (P < 0.05) (Table 5).

**Table 5:** Musculoskeletal health measured by DXA scan

Group	Group			df	F	P values
	I (n = 718)	II (n = 1158)	III (n = 1122)			
Spine BMD (g/cm^2^)	1.12(0.16)	1.08(0.18)	1.09(0.19)	2, 2497	24.26	< 0.001
L14 BMD (g/cm^2^)	1.22(0.17)	1.17(0.19)	1.19(0.22)	2, 2497	17.49	< 0.001
Femur neck BMD (g/cm^2^)	0.99(0.14)	0.94(0.15)	0.92(0.15)	2, 2497	63.75	< 0.001
Femur wards BMD (g/cm^2^)	0.81(0.15)	0.73(0.15)	0.70(0.15)	2, 2497	97.95	< 0.001
Femur troch BMD (g/cm^2^)	0.86(0.15)	0.83(0.17)	0.83(0.18)	2, 2497	21.88	< 0.001
Femur total BMD (g/cm^2^)	1.05(0.14)	0.99(0.16)	0.98(0.17)	2, 2497	53.84	< 0.001
Leg lean mass (kg)	8.14(1.94)	7.85(1.83)	7.77(1.65)	2, 2497	92.35	< 0.001
Trunk lean mass (kg)	22.91(4.40)	22.66(4.49)	22.59(4.23)	2, 2497	17.08	< 0.001
Total lean mass (kg)	47.88(10.08)	46.94(9.91)	46.56(9.08)	2, 2497	64.66	< 0.001

Data are presented as mean (S.D.); Group I: 40–49 yrs; Group II: 50–59 yrs; Group III: 60–69 yrs

### Correlation between Self-Reported and Measured Physical Activity Levels

In the youngest age group (group 1), there were generally weak correlations between self-reported and measured levels of physical activity at different intensity levels, although the correlations were statistically significant (P < 0.05). The correlations were negligible (correlation coefficients < 0.1) in groups 2 and 3 (Table 6).

**Table 6:** Correlation coefficients between self-reported and measured physical activity

Group	Group			P values
	I (n = 718)	II (n = 1158)	III (n = 1122)	
Self-reported duration of walking *vs*. measured duration of physical activity at light intensity	.13	.06	.09	I: 0.001
				II: 0.056
				III: 0.002
Self-reported duration of moderate activity *vs.* measured duration of physical activity at moderate intensity	.13	.00	.03	I: < 0.001
				II: 0.946
				III: 0.333
Self-reported duration of vigorous activity *vs.* measured duration of physical activity at vigorous intensity	.15	.05	.09	I: < 0.001
				II: 0.073
				III: 0.003

Group I: 40–49 yrs; Group II: 50–59 yrs; Group III: 60–69 yrs

## Discussion

This is a large cohort study of 2,998 middle-aged adults which showed opposing trends in their self-reported and measured levels of physical activity. This suggests that the participants over-report their activity level as they age, especially in low and moderate intensity activities. Their physical activity level, as measured by accelerometry, actually decreased significantly with age, and the same pertained to their musculoskeletal health.

The strength of the study included the use of an algorithm which allowed us to calculate the loading dose of physical activity, as compared to most previous work which only reported the duration of physical activity taken by the participants (Colley et al. 2018; Dyrstad et al. 2014; Hagstromer et al. 2010). The loading dose is clinically important as it reflects the physiological response of bone and muscle to mechanical loading. Previous studies demonstrated that loading dose of physical activity at moderate-to-vigorous intensity was significantly associated with muscle strength and bone density in middle-aged adults (Chahal et al. 2014; Luo et al. 2018).

A limitation of this study is that the different assessments had been collected at different time points. There is also a limitation in the measurement of physical activity using wrist-worn accelerometer, which may not be able to reliably capture activities such as cycling, swimming, strength training, and heavy manual work (Skender et al. 2016; Troiano et al. 2012). It is possible that body weight, height, or BMI could be potential confounding variables, but our study did not find any significant differences in these variables among the three groups. There might be other potential confounders, such as participants’ fitness levels, that could have influenced their perceived and actual physical activity levels. However, fitness level was not included in our analysis as such data were not available from UK Biobank.

The decrease in fitness with ageing leads to an increase in the relative exercise intensity (which is the percentage of the maximum capability of work). This may cause participants to perceive that they need extra effort to pursue the same physical activity (Troiano et al. 2008). Thus, the self-perceived exercise level may be an overestimate of the true exercise level. The accelerometer algorithm used in this study is a direct measurement of the mechanical loading which is important for maintaining and promoting musculoskeletal health. It is not the same as the commonly used MET (metabolic equivalent) measurements and may therefore not directly represent the absolute exercise intensity. The self-reported questionnaire and the accelerometer measurement represent different physiological domains of exercise intensity.

The overestimation of the self-perceived physical activity level may also be caused by the ambiguity in the definition of various types of activities (Durante and Ainsworth 1996). Although examples and physiological cues were provided to participants when they were completing the questionnaire, participants may still have to apply their own interpretation of intensity definition in their answers dependent upon their experience, level of fitness, age, or other demographic variables (Troiano et al. 2012). It is also possible that the increase in self-reported physical activity may be attributed to the positive self-perception of ageing in middle adulthood (Andrews et al. 2017; Beyer et al. 2015). The positive self-perception may be explained by a number of psychological and emotional factors such as sense of well-being, positive feelings or self-esteem, fear of illness or ageing, and wanting to promote a healthy old age (Kelly et al. 2016).

The current study clearly shows that self-reported physical activity levels among middle-aged adults increase in successive age groups. On the contrary, results from previous studies showed that self-reported physical activity including walking, vigorous activity, and moderate activity decreased with age during middle adulthood (Hagstromer et al. 2010). It is difficult to explain this discrepancy as different physical activity questionnaires were used in the current and previous studies. However, the current study is in line with previous findings that the self-reported physical activity level is generally greater than the measured physical activity level (Dyrstad et al. 2014; Hagstromer et al. 2010). It has been suggested that both self-reported questionnaire and accelerometry should be employed in the assessment of physical activity behaviour as they capture different aspects of activities (Troiano et al. 2012). Indeed, the self-reported questionnaire data of the current study showed that middle-aged adults are trying to shift their physical activity into low and moderate intensity range, and to those activities that are easily linked to their daily life (e.g. walking and light DIY). On the other hand, the accelerometry data showed that the shift in physical activity behaviour may not be as successful as the participants would have thought. Contrary to the questionnaire data, accelerometry would not be suitable to reveal the nature of the physical activity they were engaged.

This study showed that there were weak or negligible correlations between self-reported and measures of physical activity level. The correlations were almost non-existent in the older age groups. These findings are consistent with the observation of the opposing trends of self-reported and measured levels, and the notion that the two measures may capture different aspects of physical activities as discussed above.

Middle adulthood is the stage that musculoskeletal health starts to show significant deterioration. It is important that physical activity is maintained or even increased during this period to minimise the likelihood of deterioration in later life. However, findings of the current study suggest that the middle-aged adults tend to believe that their physical activity level is maintained or even increased, although their actual physical activity level is decreasing. This may cause them to be less responsive to advice and guidelines on becoming more active. It is also interesting to note in the current study that middle-aged adults reported that they spent more time in everyday activities such as walking, walking for pleasure, and light DIY, rather than activities such as strenuous sports, swimming, fitness, and bowling. This finding suggests that it may be easier for middle-aged adults to increase their physical activity level through everyday activities where they spend most of the time. Future studies should investigate how accelerometer-based measures may promote the awareness of middle-aged adults about their physical activity level so as to prevent the decline in musculoskeletal health as observed in this study.

## Conclusion

In conclusion, middle-aged adults over-report their physical activity level as they age, while their objectively measured physical activity and musculoskeletal health decrease during middle adulthood. They should be aware of the difference between their perceived and actual physical activity levels, and objective measures would be useful to prevent the decline in musculoskeletal health.

## Data Availability

The datasets generated during and/or analysed during the current study may be available from the authors on reasonable request.

## Code availability

The code generated during the current study is available from the authors on reasonable request.
